# Latino family caregivers: “it’s what we do—we take care of our own”

**DOI:** 10.1093/geronb/gbag086

**Published:** 2026-05-12

**Authors:** Bianca Shieu, Daria B Neidre, Lixin Song, Roxana E Delgado, M Cary Reid, Joseph E Gaugler, Carole L White

**Affiliations:** School of Nursing, University of Texas at San Antonio, San Antonio, Texas, United States; The Glenn Biggs Institute for Alzheimer’s and Neurodegenerative Diseases, University of Texas at San Antonio, San Antonio, Texas, United States; School of Nursing, University of Texas at San Antonio, San Antonio, Texas, United States; School of Nursing, University of Texas at San Antonio, San Antonio, Texas, United States; Division of Geriatrics and Palliative Medicine, Weill Cornell Medicine, Cornell University, New York, New York, United States; School of Public Health, University of Minnesota, Minneapolis, Minnesota, United States; School of Nursing, University of Texas at San Antonio, San Antonio, Texas, United States; (Social Sciences Section)

**Keywords:** Latino family caregivers, Alzheimer’s disease and related dementias, Community-Based Participatory Research, Qualitative analysis

## Abstract

**Objectives:**

To better understand the interplay of culture and family caregiving among Latinos, we explored the perceptions of caregivers of persons living with dementia on how their culture informs the care they provide.

**Methods:**

This study used a descriptive qualitative design to explore the perceptions of Latino family caregivers of persons living with dementia. A Community-Based Participatory Research (CBPR) approach underpinned the study design and execution, with a Latino Caregiver Advisory Council (LCAC) engaged in study design, recruitment, and interpretation. Individual semi-structured interviews were conducted via Zoom with Latino family caregivers of persons living with dementia from South Central Texas, in either English or Spanish. The interview guide consisted of questions about participants’ perceptions of caregiving and culture. Content analysis was used to analyze the transcripts.

**Results:**

Among 13 caregivers, we identified two overarching themes, “The Influence of Culture on Caregiving” and “Barriers to Care,” that together illustrate how cultural values serve as a profound source of both resilience and distress. We identified key subthemes related to the influence of culture (i.e., motivations for providing care, who provides this care, and where it is provided) and barriers to care (i.e., cultural beliefs about dementia, structural barriers to accessing resources, and systemic financial challenges).

**Discussion:**

Our findings indicate that culture can be a source of strength and a source of stress for Latino family caregivers. These results highlight the need for tailored caregiver support intervention programs that recognize the cultural influence shaping how families give and receive care.

Latino adults have a disproportionately higher risk for Alzheimer’s disease and related dementias (ADRD) than non-Latino White adults, and a rapidly increasing prevalence projected to reach 3.5 million by 2060 (Wu et al., 2016). Family and other unpaid caregivers predominantly care for people with dementia, frequently undertaking intensive responsibilities with limited training and support; within this broader context, literature highlights how Latino families navigate these heavy demands (Alzheimer’s Association, 2025). Compared with non-Latino White caregivers, Latino caregivers tend to provide more hours of care, perform more activities of daily living, and are less likely to use formal support services (AARP Family Caregiving and National Alliance for Caregiving, 2025). Although many Latino caregivers report that caregiving provides a strong sense of purpose, they also face substantial physical and emotional strain (Cabrera et al., 2021). Latino caregivers often report poorer health outcomes; for example, dementia severity has a direct relationship with caregiver burden, and caregiver comorbidities are associated with more depressive symptoms (Luchsinger et al., 2015; Rote et al., 2019). Culturally-rooted values, such as strong intergenerational obligation, which foster a preference for family-based care and influence when and how formal support is sought, shape these patterns (Arévalo-Flechas et al., 2014).

Prior research and observed differences in caregiving patterns indicate that culture shapes how families understand, assume, and enact dementia care responsibilities (Dilworth-Anderson et al., 2004; Pinquart & Sörensen, 2005; Zarzycki et al., 2022). These cultural values, beliefs, and traditions influence the motivation for providing care, who provides it, and where it is provided (Greenwood & Smith, 2019; Stanford University, 2024; Zarzycki et al., 2023; Zarzycki et al., 2022). To situate these dynamics within broader cross-cultural theories of kinship and care, such as the sociocultural stress and coping model, it is essential to recognize that caregiving is not merely an individual task, but a structurally and culturally embedded family system (Knight & Sayegh, 2010). Within a cross-cultural framework, key concepts include *familismo* (i.e., strong identification with and attachment to family) and *respeto* (i.e., the belief that all persons deserve respect), which reinforce a preference for family care over formal support services. Cross-cultural literature suggests that these kinship values have dual functions. On one hand, they act as powerful cultural assets that foster kinship solidarity, resilience, and positive aspects of dementia care, such as a deep sense of purpose and reciprocal love (Roth et al., 2015). On the other hand, when these strong internalized obligations collide with the demands of dementia, caregivers may experience amplified burden.

Culture also shapes who assumes this care. Latino caregivers are primarily female (61%) compared with non-Latino White caregivers (54%) (Parker & Fabius, 2022). Historically, this disparity has been linked to traditional gender socialization, including the concept of *marianismo*—the cultural expectation that a woman places the needs of the family above her own (Mendez-Luck & Anthony, 2016). However, cross-cultural literature emphasizes the need to distinguish between cultural burden and structural burden (Losada et al., 2010). While cultural norms like *marianismo* may predispose Latino women to caregiving, the assumption of the caregiving role is also driven by broader structural and socioeconomic factors that position women as default unpaid caregivers (Cabrera et al., 2021; Vega et al., 2017). Thus, their experience of burden is heavily compounded by structural barriers, including systemic inequities, economic hardship, and a lack of culturally tailored resources. Furthermore, as more Latinas enter the formal workforce, caregiving responsibilities are increasingly diversified, disrupting traditional paradigms (Cabrera et al., 2021; Vega et al., 2017). Similarly, culture influences where care occurs. Compared with non-Latino White caregivers, Latino caregivers more often live with their care recipients, potentially reflecting the multigenerational living common among Latino families (Guzman & Skow, 2019).

These characteristics of Latino caregivers highlight challenges that Latino families experience in providing dementia care. Several studies acknowledge the influence of culture on family caregiving experiences, but fall short in explicitly identifying the specific cultural facilitators and barriers that families encounter when navigating the healthcare system and performing daily care duties (Garcia et al., 2024; Jaldin et al., 2023; Vila-Castelar et al., 2022). As such, this study explored the experiences of Latino families caring for a family member living with dementia and their perceptions about how their culture may influence their caregiving. Explicitly identifying these cultural facilitators and barriers prevents poor alignment between interventions and caregivers’ lived realities and the underutilization of resources despite high need.

This study used the term “dementia” to describe a range of conditions that include, most commonly, Alzheimer’s disease, vascular dementia, and Lewy body dementia, as well as other neurocognitive disorders (Alzheimer’s Association, 2025). Additionally, we followed our Latino Caregiver Advisory Council’s recommendation to use “Latino” consistently throughout the study to denote persons living in the United States whose origins trace to Spanish-speaking regions of Latin America and who identify as Hispanic/Latino.

## Methods

### Design

This study used a descriptive qualitative design to explore Latino family caregivers’ perceptions of how their culture informs the care they provide. A Community-Based Participatory Research (CBPR) approach underpinned the study design and execution, with a Latino Caregiver Advisory Council (LCAC) providing oversight (Collins et al., 2018). This study represents the first phase of a larger research project to culturally adapt and examine the feasibility of a psycho-educational intervention for Latino family caregivers (White et al., 2025; White et al., 2026).

### Setting

This community-engaged study was conducted within the Caring for the Caregiver Program at the University of Texas at San Antonio School of Nursing (UT San Antonio SON). This study was approved by the University Institutional Review Board at UT San Antonio. All caregivers enrolled in the study provided informed consent.

### Participants

#### Latino caregiver advisory council

The LCAC provided oversight and guidance to the research project. The council comprised Latino family caregivers recruited through the UT San Antonio SON Caring for the Caregiver Program, as well as members of the research team.

#### Qualitative study participants

Participants were caregivers of individuals diagnosed with a form of dementia, who met the following criteria: (1) self-identify as Latino, (2) 18 years of age or older, (3) assisting with at least two instrumental activities of daily living (IADL) or one activity of daily living (ADL), and (4) able to speak and read English or Spanish.

### Study procedures

*LCAC Involvement*: Guided by a CBPR framework, we established the LCAC to foster shared decision-making and co-learning between community members and academic researchers. The LCAC comprised equal representation of Latino family caregivers (*n* = 5) and research team members (*n* = 5). Caregiver members included spouses (*n* = 3), a mother (*n* = 1), and an aunt (*n* = 1); two possessed prior experience on caregiver councils, and one facilitated a support group. The research team included three faculty members with expertise in family caregiving, a research coordinator, and a research assistant (two of whom identified as Latino).

LCAC members reviewed the study aims, provided verbal consent, and received a stipend for their time and expertise. The council met monthly for 1.5 hours via Zoom to engage in all phases of the study, including questionnaire design, material translation, recruitment, and data analysis. Operationalizing the CBPR approach, LCAC input led to concrete refinements in the study protocol. For instance, members revised the recruitment flyer to use more culturally resonant language regarding “familismo.” Additionally, during the analysis phase, the council provided context for specific themes, clarifying that certain caregiving behaviors were viewed as distinct cultural duties rather than burdens.

*Participant Recruitment and Interviews*: The study was advertised via social media posts in Spanish and English, and targeted emails to caregivers who self-identified as Latino in a family caregiver program database. Recruitment activities also targeted community-based organizations, including the local chapter of the Alzheimer’s Association. Caregivers who expressed interest in the study contacted the research assistant, who screened them for eligibility. If eligible, the caregiver received further information about the study, and an information sheet was emailed to them. The research assistant informed the participants about the confidentiality of the interview and, if they were interested in participating, provided written informed consent via the Research Electronic Data Capture (REDCap) management system.

Interviews were conducted in English or Spanish, according to the participant’s preference. The LCAC developed a semi-structured interview guide, with forward and back translation undertaken by an LCAC caregiver and two bilingual study team members. Questions used in the guide included: “What are the things that are most important to you as a Latino family caregiver?” “Describe your experiences of being a family caregiver and how you think your culture has influenced you taking on this role,” and “How did you learn how to take care of your family member?” See Table 1 for the complete interview guide.

**Table 1:** Interview guide.

Question #	Question
**1**	Describe ways that you identify with your Latino/Hispanic culture. What are some of the things that are most important to you in your culture?
**2**	Describe your experiences of being a family caregiver and how you think your culture has influenced you in taking on this role.
**3**	How did you learn how to take care of your family member? In learning, was there anything unique about your experience in regard to your Latino/Hispanic culture?
**4**	What are some of your biggest concerns about the health and health care for your family member and how he/she is doing in terms of his/her quality of life?
**5**	What about your own health? How do you feel that caregiving is affecting your health and well-being?
**6**	What are some of the challenges you experience in providing care? Are there aspects of caregiving that you find particularly difficult?
**7**	Are there things that you feel need to be improved related to healthcare and caregiving for Latino/Hispanic caregivers? Are there resources that you think would make caregiving easier?

Eligible caregivers participated in a single Zoom-based, semi-structured interview focused on their personal experiences as Latino family caregivers of individuals living with dementia. Each interview was audio-recorded and transcribed verbatim for analysis. Trained members of the research team conducted all interviews to provide uniformity and consistency in the interview process. The initial part of the interview focused on demographic questions and the assistance caregivers provide, including the 6-item ADLs and 8-item IADLs questionnaires. Participants answered open-ended questions about their caregiving experiences, including aspects they perceived were unique to their Latino culture and customs. The interview sessions concluded upon completion of the interview guide and termination of the recording.

#### Data analysis

Demographic and survey data (ADL/IADL) were analyzed using descriptive statistics (e.g., frequencies) to describe the sample.

Interviews were transcribed verbatim using Zoom’s built-in live transcription feature. Qualitative data were analyzed using inductive content analysis, employing a constant comparative approach throughout coding and theme development (Gray et al., 2017). To begin, three research team members—including two with qualitative expertise—independently reviewed and coded the first transcript. The team then met to discuss these initial codes and collaboratively developed a comprehensive codebook documenting all code definitions, analytical decisions, and illustrative examples. For all subsequent transcripts, two team members independently reviewed and coded the data. Any discrepancies between the reviewers were reconciled through collaborative discussion, with a senior investigator resolving any persistent disagreements.

To transition from individual codes to overarching themes, the research team iteratively grouped related codes to identify patterns regarding the perceptions of Latino family caregivers and culture. The resulting themes and representative quotes were then shared with the LCAC. Seeking the LCAC’s input on interpreting the data helped finalize the findings and significantly enhanced their confirmability. Finally, no new patterns or significant themes emerged during the coding of the last two interviews, indicating that data saturation had been reached (Creswell, 2011).

## Results

### Participant demographics

The caregiver participants comprised 13 Latino family caregivers from South Central Texas, consisting of spouses (*n* = 5) and adult children (*n* = 8). Twelve interviews were conducted in English, and one was conducted in Spanish. Our family caregiver program database serves as our primary recruitment source. While the majority of these caregivers identify as Hispanic/Latino, many are second-, third-, or later-generation individuals born and raised in the United States. Consequently, their preferred language is predominantly English. Although we did not formally collect data on the exact length of time participants have resided in the US, the majority of the interviewed caregivers (*n* = 7) were US-born. Detailed demographic characteristics by participant are presented in Table 2. Participant age ranged from 43 to 80 years. Most participants self-identified as Latino White (*n* = 11), with two identifying as multiracial. The participants were predominantly of Mexican, Mexican American, or Chicano heritage (*n* = 11), while two participants identified as Puerto Rican. Educational attainment was varied and included primary-level schooling (*n* = 1), a secondary degree or equivalent (*n* = 3), some college attendance (*n* = 4), and a bachelor’s degree or higher (*n* = 5). The majority of participants were married (*n* = 9), three were widowed, and one was never married. When asked if they had sufficient money to meet basic living needs, most caregivers (*n* = 9) reported that they did, whereas four indicated they rarely or never had enough. The assumption of caregiving responsibilities also coincided with changes in employment for several participants: six transitioned from full-time employment to reduced hours or unemployment, one moved from unemployment to paid work, two reported no change, and four were not in the workforce when caregiving began.

**Table 2:** Demographic characteristics.

xCaregiver ID	Gender	Age	Ethnicity	Education level	Employment status	Sufficient money to meet basic living needs	Recipient of care	Live with recipient of care	Number of ADLs	Number of IADLs
CG01	Male	56	Mexican American	Bachelor’s degree	Retired	Always	Mother	Yes	3	7
CG02	Female	42	Mexican American	Bachelor’s degree	Stopped work	Always	Mother	Yes	4	8
CG03	Female	65	Mexican American	Primary school	Unemployed	Some of the time	Husband	Yes	1	8
CG04	Female	58	Mexican American	Bachelor’s degree	Employed full-time	Always	Mother	Yes	1	6
CG05	Male	81	Mexican American	Secondary school	Retired	Always	Wife	Yes	5	8
CG06	Female	79	Mexican American	Secondary school	Retired	Always	Husband	Yes	5	8
CG07	Female	66	Mexican American	Secondary school	Stopped work	Never	Father	Yes	5	8
CG08	Female	42	Mexican American	Bachelor’s degree	Employed full-time	Always	Mother	No	0	8
CG09	Female	63	Mexican American	Bachelor’s degree	Stopped work	Always	Mother	No	5	8
CG10	Female	63	Mexican American	Secondary school	Employed full-time	Some of the time	Mother	Yes	4	8
CG11	Female	64	Puerto Rican	Secondary school	Retired	Always	Mother	Yes	4	8
CG12	Male	81	Mexican American	Secondary school	Stopped work	Always	Wife	Yes	4	8
CG13	Female	54	Puerto Rican	Secondary school	Stopped work	Never	Husband	Yes	2	8

*Note.* ADL = activities of daily living; IADL = instrumental activities of daily living.

### Themes

Based on our qualitative interviews, we organized our findings into two themes: The Influence of Culture on Caregiving and Barriers to Care (see Table 3).

**Table 3:** Themes and subthemes.

Themes	Subthemes
**The influence of culture on caregiving**	Motivations for providing care (i.e., Latino identity and family) Who takes on this care Where the care is provided
**Barriers to care**	Cultural beliefs about dementia Structural difficulties accessing resources (i.e., health care access, health insurance, transportation, and education materials) Systemic financial challenges

#### The influence of culture on caregiving

Three subthemes were identified under this broader theme: (1) motivations for providing care, (2) who takes on this care, and (3) where the care is provided.

The first subtheme, ***Motivations for Providing Care***, was deeply intertwined with Latino identity and family. One participant stated, “culturally, we take care of our loved ones because they are our loved ones” (CG05). This role was often a source of immense pride, with caregivers viewing their cultural upbringing as giving them an “upper hand through a legacy of compassion passed down through generations” (CG01). The following quotation encapsulates this sense of duty toward caring for family members: “It’s what we do, we take care of our own” (CG05). A central principle expressed by participants was: “Family first…Caring for loved ones is a non-negotiable responsibility” (CG02). Participants consistently emphasized the importance of their familial bonds, describing their families as “loyalty, united family” (CG08) and “a very close-knit family” (CG01). This sentiment was echoed by another participant who stated simply, “My family is important” (CG02).

This cultural emphasis on duty to care, however, could also lead to hardships for caregivers. In several interviews, caregivers discussed a cultural belief that asking for help from formal care services or extended family equated to failing in their familial obligations, with one noting, “I’m inadequate if I asked for help. I’m not doing my… my daughter duties” (CG02). This extends to suppressing personal needs, with the sentiment that “There’s no depression in Latino. There’s no anxiety in Latino. You deal with it!” (CG07).

The second subtheme related to ***Who Takes on This Care*.** This profound sense of family duty often determined how care was provided, with responsibilities heavily shaped by family roles, primarily defined by gender, birth order, and cultural norms. A prominent finding was the perception of caregiving as a woman’s role, with participants stating it has “always been known that the woman is going to provide all the services” (CG07) and “caregiving is considered a lady’s job” (CG12). This created situations where female caregivers felt male siblings were exempt from duties, even when their help seemed more appropriate, such as one participant questioning “why my brother wasn’t attending our father’s urology appointments” (CG02). Moreover, birth order significantly influenced the assumption of the caregiving role. As one caregiver stated, “I was the oldest born” (CG01), while another stated. “[I felt] pressure to care for everybody, specifically because I am the eldest daughter” (CG02). Furthermore, these family-driven responsibilities were often compounded by the need to care for multiple people simultaneously. Many participants found themselves in complex arrangements, such as one caregiver stating, “looking after two parents while also financially supporting my wife in another country” (CG01). Some found themselves in a “sandwich generation role, caring for both a disabled child and an aging parent” (CG02).

The third subtheme addressed ***Where the Care is Provided*.** A strong preference for informal, family-based care over formal services was a predominant finding, rooted in the core belief that “no one’s going to take care of them as well as we can” (CG05). This belief was coupled with significant distrust of formal care systems, as one caregiver stated, “I don’t want strangers coming in my home” (CG01). This apprehension grew from fears that accepting outside help could lead to a loved one being “forcibly put in a nursing home” (CG01) or from “suspicions of misconduct, such as theft” (CG05). Consequently, “placing a family member in a nursing home is overwhelmingly viewed as a last resort” (CG05), a decision made only when the family is no longer physically able to provide care. This reluctance was further solidified by intergenerational promises, with one caregiver recalling “my mother’s plea not to put her in a home, a value bred into us” (CG07). To uphold this commitment, families often created their own support systems, such as “organizing rotating shifts among siblings to manage the caregiving responsibilities within the family unit” (CG06).

#### Barriers to care

While cultural values heavily influenced caregiving approaches, participants also described significant systemic and structural barriers that impeded their access to formal services. It became clear during the analysis that the underutilization of support was not solely a reflection of cultural preference but was heavily driven by external constraints. Three subthemes emerged under barriers to care: cultural beliefs about dementia, structural difficulties accessing resources, and systemic financial constraints.

The first was ***Cultural Beliefs about Dementia***. A significant barrier to understanding and addressing dementia was the belief that its symptoms were a normal part of aging. For instance, one caregiver frequently reported that “initial signs were dismissed as just their old age” (CG07), with another participant noting that “even a doctor initially attributed her mother’s symptoms to age” (CG08). This perception connects to a broader, self-identified lack of awareness within the Latino community, with one caregiver stating, “still Latino people, still to this day, don’t know that it’s a disease and you need to get some help” (CG07). This could lead to a delayed diagnosis, with one caregiver stating, “We first thought our loved one was just acting weird before receiving a formal explanation like mild dementia” (CG03). Furthermore, a dementia diagnosis could carry a significant stigma, viewed by some as meaning “you’re not like a complete person anymore, creating a desire to keep the condition hidden from others” (CG 02).

The second barrier was ***Structural Barriers to Accessing Resources***. This broad theme includes four distinct systemic challenges: health care, health insurance, transportation, and educational materials. A lack of culturally and linguistically concordant care significantly hindered access to available health care services. For example, a caregiver shared “no one at the adult daycares can speak Spanish to the patient” (CG05). Geographic location also played a crucial structural role, with a caregiver stating, “We feel very isolated and lack the resources in smaller, rural communities compared to larger cities” (CG04). To bypass these local systemic roadblocks, some families even turned to cross-border solutions, such as “seeking doctors in Mexico when local care is inaccessible” (CG10) or “relying on private caregivers to get much-needed respite” (CG06). When caregivers managed to connect with specialized professional care providers, such as a geriatric doctor, a caregiver described “the help is huge and tremendous” (CG08), highlighting the profound difference that access to health care can make. On the other hand, some caregivers noted “we are outliers because we have access to good health professionals and facilities such as the Veterans Administration, being a critical resource for in-home nursing and physician visits” (CG01 and CG06).

Navigating health insurance was another bureaucratic barrier to accessing resources. For some, “consistent insurance coverage provides a stable foundation for accessing care without major issues” (CG01). However, many faced significant systemic hurdles, including the transition from Medicare to Medicaid. This process involved extensive paperwork and logistical challenges, including “moving the care recipient to a different facility once one form of coverage is exhausted” (CG12).

Transportation was another access-related factor that affected care. One caregiver noted that “caregivers often became the sole providers of transport for all their loved ones’ appointments” (CG06). The lack of personal or accessible transportation prevented many caregivers from accessing additional resources like “support group meetings” (CG08), with one participant pointing out that “unreliable transportation and a lack of community transit services make it difficult to go anywhere” (CG01). Similarly, another participant said that “even when formal help like social services is available, long waitlists create an additional burden when the need for transportation is immediate” (CG12).

Caregivers consistently reported a significant lack of educational resources, including those tailored for Spanish-speaking caregivers, which created feelings of isolation and left them unprepared for their roles. Many reported lacking formal training, stating, “there’s no training… we cook, clean, and care for our loved ones” (CG07) and “it can take a long time just to learn what limited help was available” (CG12). “There is a strong desire for more structured support, such as aftercare programs, grief counseling, and support groups to combat loneliness and prepare for life after caregiving” (CG07).

The third barrier to care was ***Systemic Financial Challenges*.** Caregiving often forces individuals into unemployment or underemployment, leading to “immense stress about whether they will make ends meet” (CG13). Many caregivers reported “struggling financially” (CG01), “paying significant amounts out-of-pocket” (CG05), and having to make impossible economic choices between “a tank of gas to drive to [the name of a city] or paying for a doctor’s visit” (CG01). Furthermore, navigating insurance and aid systems presented a significant hurdle, with some stating they found “we don’t qualify for aid because we are not in the right financial category” (CG02) or “because our loved one lacks the work history for Medicare” (CG10), highlighting how employment history and citizenship status directly restrict care access. On the other hand, some caregivers described “families pooling money among siblings to pay for private help” (CG10) or “relying on private caregivers for respite” (CG06).

Many caregivers were “forced to retire early” (CG05), quit their jobs, or relocate to provide full-time care, with one participant explaining she had to “quit my job, sell the house and pay off all the bills so I could be able to care for him… without an income” (CG07). This complete “withdrawal from the workforce could lead to long-term financial dependence” (CG07) on the care recipient’s income and prevent caregivers from caring for themselves, as “we are no longer employed” (CG13). In contrast, one caregiver with a more flexible work situation stated, “being self-employed or holding a supervisory role, I was better able to manage my dual responsibilities without leaving my job” (CG01).

## Discussion

This study demonstrates the value of a CBPR framework. This approach elevates our theoretical understanding of cultural caregiving while ensuring that future interventions are developed with the community rather than solely for them (Collins et al., 2018). Through this engagement, caregivers described caregiving as a core expression of identity and belonging, “it’s what we do,” grounded in familismo and intergenerational obligation. This pride and meaning coexisted with significant emotional, physical, and financial strain, compounded by a profound sense of stigma. Notably, our participants’ expression of this stigma—specifically the perception that the care recipient is “no longer a complete person”—diverges from some prior studies on Latino dementia caregiving, which often highlight a more normalizing or spiritually accepting view of cognitive decline (Cabrera et al., 2021; Neidre et al., 2025). This unique finding may reflect our highly acculturated, predominantly US-born sample, whose interpretations of dementia may be more heavily influenced by broader societal values placed on independence and cognitive autonomy. This stigma, along with delayed recognition of dementia and limited access to linguistically and culturally concordant services, further complicated the caregiving experience. Consistent with Garcia et al. (2024), our findings suggested that a strong preference for family-based care, mistrust of strangers, and fear of nursing home placement made formal support a last resort. These dynamics help explain the underutilization of services despite high need.

Our findings extend longstanding evidence that culture shapes who provides care, motivations for care, and care settings (Dilworth-Anderson et al., 2004; Greenwood & Smith, 2019; Pinquart & Sörensen, 2005). Consistent with findings from previous studies, women, especially the oldest daughters, disproportionately assumed caregiving roles, reflecting marianismo and deep-rooted gendered expectations, as well as higher female involvement in unpaid care (Mendez-Luck & Anthony, 2016; Parker & Fabius, 2022). Multigenerational households and close-knit family structures facilitated intensive, home-based care (Arévalo-Flechas et al., 2014; Guzman & Skow, 2019). Furthermore, this study’s findings add nuance by detailing how help‑seeking as a sign of weakness, mental health suppression, and fear of institutionalization concretely shape day‑to‑day decisions to reject or delay formal services, echoing cultural duty models and recent Latino‑focused analyses of mistrust and preference for family care (Gelman, 2014; Martinez & Acosta Gonzalez, 2022; Mendez-Luck & Anthony, 2016; Molina et al., 2019; Zarzycki et al., 2022).

This study identified barriers, such as normalizing early symptoms as just aging, language barriers, rural isolation, transportation gaps, and financial strain, that map onto and help explain documented challenges in caregiver health and service utilization among Latino families (Luchsinger et al., 2015; Rote et al., 2019; Wu et al., 2016). Recent qualitative syntheses and empirical studies on cultural motivations and caregiving among Latino families similarly emphasize the importance of cultural identity, duty, and love as motivators, while highlighting gaps in dementia literacy and culturally safe care (Garcia et al., 2024; Jaldin et al., 2023; Neidre et al., 2025; Vila-Castelar et al., 2022; Zarzycki et al., 2022).

Our findings of rotating sibling shifts, cross-border care-seeking, and selective reliance on Veterans Affairs services are not unique to Latinos in the United States; they resonate strongly with the experiences of ethnically diverse and minoritized caregivers in other high-income countries. For instance, research among culturally and linguistically diverse (CALD) populations in the UK, Australia, and Canada demonstrates that these families frequently navigate similar structural inequalities such as language barriers, culturally incongruent dementia services, and financial strain by relying heavily on transnational care networks, community pooling, and intensive kin support (Boughtwood et al., 2011; Kenning et al., 2017; Mukadam et al., 2011). Situating our findings within this broader cross-cultural literature highlights that while specific cultural nuances may vary, the systemic marginalization of, and subsequent resilient strategies employed by, minority caregiving families represent a shared global phenomenon.

Finally, culture-driven motivation sustained caregiving in the face of hardship, but it also reinforced gendered labor, delayed help-seeking, and self-silencing about stress and depression. This tension suggests that a culturally resonant reframing is needed: seeking support can be framed as an act of responsibility that preserves dignity and fulfills family duty, rather than as a failure. Such reframing may be significant for women navigating marianismo norms and for families equating institutional care with abandonment.

### Limitations

The findings from this study should be interpreted in light of several limitations. First, consistent with the principles of qualitative trustworthiness, this study prioritized a deep exploration of lived experiences over statistical generalizability. Because the sample was drawn exclusively from South Central Texas and was primarily Mexican American (with two Puerto Rican participants), the transferability of these findings to other Latino subgroups, regions, or Spanish dialects should be considered in light of this specific context. Furthermore, there is a lack of granular data regarding participants’ specific acculturation levels, generational status, and mixed-heritage backgrounds. Latino culture is not monolithic; an individual raised in their country of origin who later migrates to the US experiences cultural expectations and family dynamics differently than someone born and raised in the US. Although we know the majority of our participants were US-born and preferred to communicate in English, we did not collect data on the exact length of time immigrant participants had resided in the US. Consequently, a key implication for our analysis is that our findings likely reflect a more acculturated, predominantly English-speaking perspective. The caregiving experiences and cultural themes identified here may differ significantly from those of recent immigrants or monolingual Spanish-speaking Latino families. Second, voluntary participation may have introduced selection bias, potentially favoring caregivers who were more comfortable sharing their personal experiences. Finally, isolating what constitutes a uniquely “Latino” cultural value presented a significant analytical challenge. Because our participants live in the US and are highly acculturated, their caregiving approaches inevitably reflect a hybrid of traditional Latino heritage and assimilated non-Latino US cultural norms. Participants sometimes struggled to explicitly articulate which cultural paradigm drove their behaviors, blurring the lines between traditional expectations, assimilated practices, and individual preferences. While consistent patterns across interviews suggest that Latino heritage remained a pervasive factor, our analysis must be understood as capturing a blended cultural experience rather than a singular, traditional framework.

### Implications

A strength of this study is that all aspects were overseen by an LCAC. Findings have direct implications for program design and implementation: integrating cultural elements need not be complex, but it does require a shift from top-down instruction to collaborative partnership with caregivers. Programs should be sensitive to cultural context and the pride associated with family caregiving. An effective intervention supplements and supports caregivers’ efforts, rather than replacing them or dictating actions, by respecting lived experience, honoring expertise, and providing resources that empower the family. These qualitative insights align with recent syntheses on intervention design, which also call for co-designed frameworks that integrate cultural values (Neidre et al., 2025). Framing support as walking the journey with them can honor the cultural mandate to care for our own while mitigating burden.

### Conclusion

Guided by a CBPR framework, this study reveals the complex duality of Latino caregiving: culture acts as both a source of resilience and a barrier to formal support. While values provide a deep sense of purpose (i.e., “it’s what we do”), they can also lead to isolation and financial strain when coupled with systemic distrust. To address the growing burden of dementia in this population, future interventions must move beyond linguistic translation to cultural adaptation. Policymakers and clinicians must partner with communities to reframe external support as a means of preserving the family unit rather than replacing it.

## Data Availability

The qualitative datasets (e.g., de-identified transcripts) generated and analyzed during the current study are available from the corresponding author upon reasonable request, subject to ethical constraints and participant confidentiality. As this is a qualitative study, it was not preregistered.
